# Reflection and Experimental Rigor Are Our AiMS: A New Metacognitive Framework for Experimental Design

**DOI:** 10.1523/ENEURO.0333-25.2025

**Published:** 2025-10-24

**Authors:** Taralyn Tan, Xiuqi Li

**Affiliations:** ^1^Office for Graduate Education and Department of Neurobiology, Harvard Medical School, Boston, Massachusetts 02115; ^2^Department of Cell Biology, Harvard Medical School, Boston, Massachusetts 02115

**Keywords:** experimental design, generative AI, metacognition, rigor

## Abstract

Experimental design is a core competency of scientific training with profound implications for research rigor and reproducibility. Yet, trainees often receive minimal guidance to structure their thinking around experimental design. Metacognition—reflecting on one's own thinking—offers a powerful tool to strengthen reasoning in this process. Here, we present the AiMS framework, which adapts the classic plan–monitor–evaluate cycle of metacognition to the context of experimental design. This framework emphasizes three iterative stages—Awareness, Analysis, and Adaptation—that scaffold reflection on an experimental system defined by its Models, Methods, and Measurements and evaluated through Specificity, Sensitivity, and Stability. We illustrate application of the AiMS framework through an interactive neuroanatomy case study and provide a structured worksheet to guide readers in applying it to their own experiments. We also highlight how the framework can assist researchers in organizing their ideas for research proposals and explore the responsible use of generative AI as a metacognitive partner that supports structured reflection without supplanting original intellectual contributions. The AiMS framework complements other principles and practices of rigor by foregrounding deliberate reasoning about assumptions, vulnerabilities, and trade-offs. Our goal is to provide practical tools that foster rigor, creativity, and adaptability in the design of biological experiments, supporting both trainees and their mentors in cultivating reflective scientific practices.

## Significance Statement

Rigor in experimental design is essential for neuroscience and the life sciences more broadly. The AiMS framework provides a structured approach to reflection, helping researchers to think more deliberately about their design choices, identify underlying assumptions, and assess potential vulnerabilities. By emphasizing metacognition, the AiMS framework promotes experimental rigor by offering practical tools for trainees designing experiments and an accessible scaffold for mentors teaching experimental design.

## Introduction

Research design is a fundamental skill for life science trainees (National Academies of Sciences, Engineering, and Medicine, 2018; Verderame et al., 2018; X. Li et al., 2025), yet it is often one of the hardest to master. Designing experiments requires moving beyond executing generic protocols to making deliberate choices about how evidence will be generated and interpreted. As early-career scientists transition from structured coursework to self-directed research inquiries, they face increased responsibility for designing experiments, though oftentimes without having been given clear guidance for making these decisions.

Metacognition—thinking about one's own thinking—offers a powerful lens for scientists to become more aware of how we design, evaluate, and refine experiments. Past work has shown that metacognitive strategies strengthen critical thinking, problem-solving, and decision-making (Tanner, 2012; Zohar and Barzilai, 2013; Stanton et al., 2021). These findings highlight a key point: structured reflection supports better decisions. In the context of experimental design, where uncertainty and multiple possible paths are inherent, metacognition can provide a practical means for scientists to pause, examine our reasoning, and respond more deliberately to unexpected outcomes. Indeed, metacognitive reflection exercises to enhance experimental design have shown promise within course-based undergraduate research experiences (McCabe and Olimpo, 2020).

In this article, we present the AiMS framework, a reconceptualization of a common metacognitive model tailored to the experimental design context. Through this framework, we aim to promote rigorous experimental design through structured reflection. We also highlight how this framework can inform research proposal writing and explore how generative AI, when used responsibly as a metacognitive partner, can support such reflection. We hope this article is of practical use to both trainees and other researchers directly, as well as to science educators and research mentors as a resource for teaching experimental design.

## Introducing the AiMS Framework: Structured Metacognitive Reflection to Enhance Experimental Rigor

**Figure 1.**
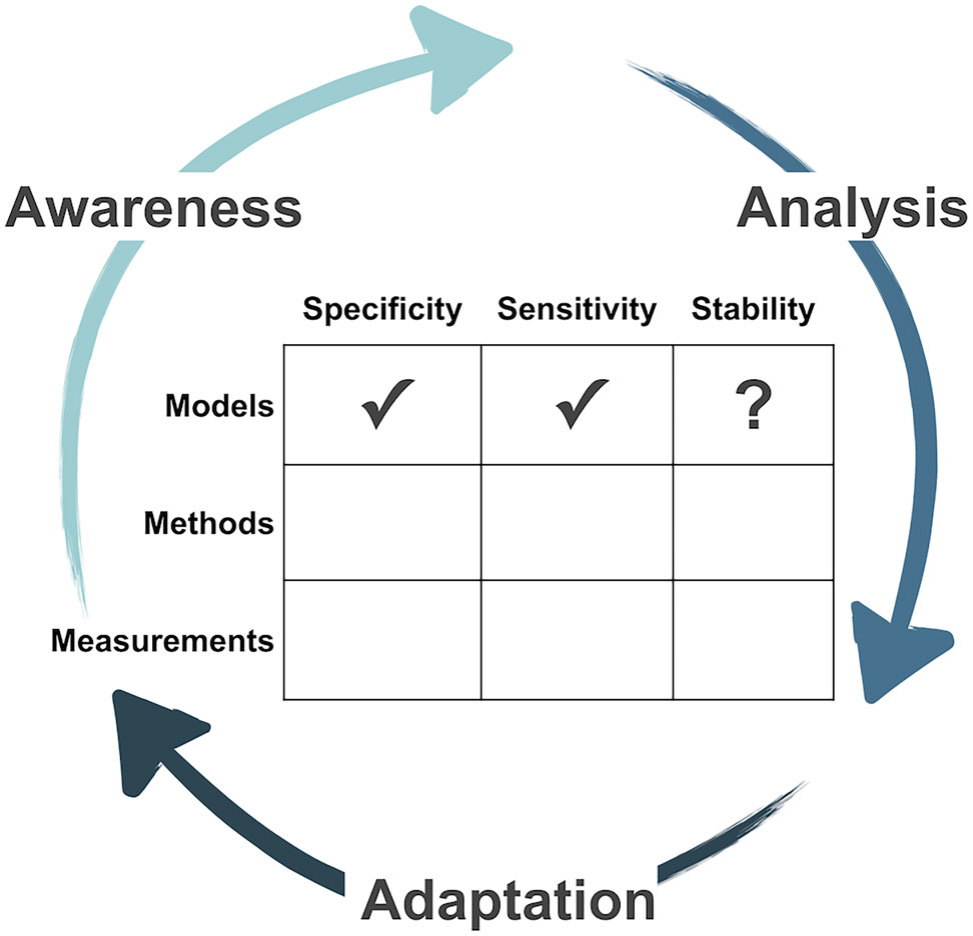
Conceptual diagram of the AiMS framework. The framework engages scientists in a metacognitive cycle adapted to experimental design. Through the Three A's—Awareness, Analysis, and Adaptation—scientists reflect on aspects of their experimental system, which is conceptualized in this framework as being composed of the Three M's—Models, Methods, and Measurements—that can in turn each be described in terms of the Three S's—Sensitivity, Specificity, and Stability. An editable blank AiMS worksheet is available as Extended Data Figure 1-1 and a completed AiMS worksheet for the neuroanatomy case study is available as Extended Data Figure 1-2.

Here we introduce the AiMS Framework (Fig. 1), through which we have adapted principles from metacognition and experimental design to scaffold thinking and promote structured reflection about experimental systems. At the heart of the framework are the Three A's: Awareness, Analysis, and Adaptation. This metacognitive cycle, adapted from the classic plan–monitor–evaluate model of metacognition that describes activities to regulate one's thinking and learning (Schraw and Moshman, 1995; Stanton et al., 2021), guides researchers to identify key features of an experimental system (“Awareness”), interrogate its limitations and possible experimental outcomes (“Analysis”), and refine the experimental design in light of this reasoning (“Adaptation”).

10.1523/ENEURO.0333-25.2025.f1-1 Figure 1-1 **Template AiMS Worksheets.** Trainees and educators can download editable worksheets that guide users through the reflective prompts of the AiMS framework. Download Figure 1-1, DOCX file.

**Figure 2.**
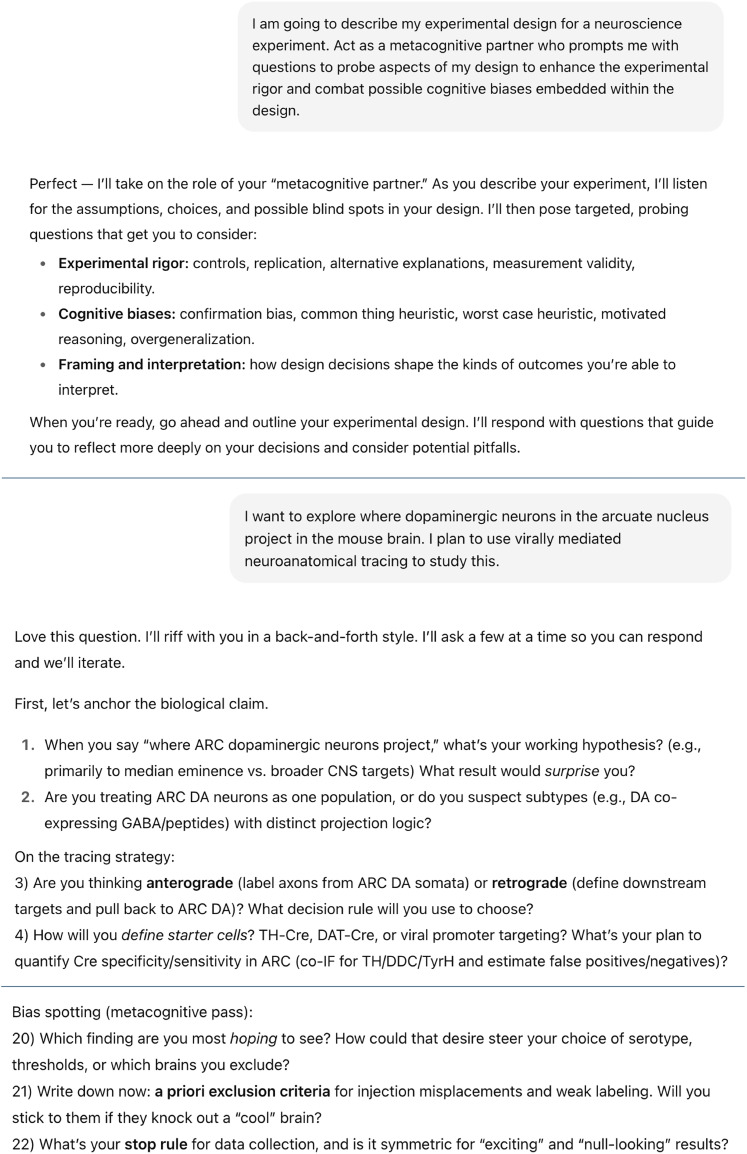
A demonstration of using generative AI as a metacognitive partner in experimental design. Screenshots depicting excerpts from an interaction with ChatGPT5 (university enterprise account) illustrate how generative AI can facilitate metacognitive reflection. Note that ChatGPT responses are stochastic and context-dependent, so this output is a representative example that is unlikely to be reproduced verbatim by other users.

10.1523/ENEURO.0333-25.2025.f1-2 Figure 1-2 **Completed AiMS Worksheet for Judy’s Neuroanatomy Case Study.** This completed worksheet summarizes the neuroanatomy case study presented in the text as a concrete example of application of the AiMS framework to an experimental question and system. An editable version of the worksheet can be downloaded and modified to provide partially worked examples in an instructional context. Download Figure 1-2, DOCX file.

To further structure thinking on experimental systems within this metacognitive cycle, we conceptualize an experimental system—everything that a scientist uses to conduct an experiment (Glass, 2014)—as consisting of the Three M's: Models, Methods, and Measurements. “Models” refer to the biological entities or subjects under study, such as in vitro models (e.g., cell culture, organoids) or in vivo models (e.g., *C. elegans*, *M. musculus*). “Methods” are the experimental approaches or perturbations applied to those models—genetic manipulations (e.g., siRNA, CRISPR-Cas9), pharmacological interventions, etc. “Measurements” are the specific readouts or data collected, whether gene expression analyses (e.g., qPCR, RNA sequencing), protein quantification (e.g., Western blot, ELISA), or metabolomics (e.g., mass spectrometry). Thinking in terms of the Three M's makes the moving parts of an experiment explicit, rather than treating the experimental system as a black box.

In turn, each of the Three M's can be described and evaluated through the lens of the Three S's: Specificity, Sensitivity, and Stability. The Three S's capture key elements of established frameworks for experimental design (Glass, 2014), highlighting properties of the system that can shape how results can be interpreted. “Specificity” asks whether the experimental system accurately isolates the phenomenon of interest. “Sensitivity” considers the ability to observe a variable of interest at the amount it exists in the experimental system. “Stability” addresses whether the experimental system remains consistent over time and under various conditions. Reflecting upon these can make visible the assumptions and trade-offs built into experimental design choices.

We note that the Three M's and Three S's are not the only way to frame an experimental system. They represent practical heuristics that are easy to learn and apply. Other approaches—such as frameworks emphasizing validity, reproducibility, or feasibility—can be equally valuable (Masca et al., 2015; Holder and Marino, 2017; Williams, 2018) and could be considered alongside the AiMS framing. While the AiMS framework was designed with wet-lab biological research in mind, we encourage readers to consider how it may be adapted to computational, theoretical, or field-based studies. Rather than prescribing specific methods or dictating a single “correct” approach, we hope to provide a conceptual scaffold for structured reflection that can be flexibly applied across research contexts.

We have developed a series of reflection prompts, organized into an AiMS worksheet (Extended Data Fig. 1-1) to guide users step by step through this framework. The next section of this article is structured as an interactive tutorial to support researchers in gaining hands-on practice applying the AiMS framework to their own experimental contexts. To ground the framework in a concrete neuroscience example, we interleave a neuroanatomy case study [based on Zhang and van den Pol (2016) and summarized in Extended Data Fig. 1-2] that illustrates how each stage of reflection can shape experimental decisions.

These materials were developed for the Community for Rigor (C4R) initiative from the National Institute of Neurological Disorders and Stroke, which is developing freely accessible, online educational modules to improve training in the principles of rigorous research. Through the accompanying online module currently in development by C4R, researchers and educators will be able to further engage with the AiMS framework, worksheets, and neuroanatomy case study presented here through interactive digital content designed to be adapted and incorporated into lab meetings or classroom settings. Further information on the C4R initiative and completed educational modules can be found on the initiative's website at https://www.c4r.io.

## Applying the AiMS Framework: An Interactive Tutorial

*Meet Judy, a first-year PhD student, who is interested in examining the function of tyrosine hydroxylase (TH)-expressing dopaminergic neurons of the arcuate nucleus of the hypothalamus (ARC)*—*best known for their role in regulating pituitary prolactin synthesis*—*in regulating energy homeostasis. Judy first seeks to characterize where in the brain the ARC–TH neurons project. Previous work on ARC–TH cells identified axonal projections to the median eminence (ME), but it is unclear whether these cells project elsewhere in the brain. The paraventricular nucleus of the hypothalamus (PVH), a region near the ARC, is important for energy homeostasis and receives axonal input from other ARC neuronal populations. Thus, Judy's working hypothesis is that the ARC–TH neurons project to the PVH in addition to the ME. She plans to conduct a neuroanatomical tracing experiment: injecting an adeno-associated virus (AAV) expressing a Cre-dependent green fluorescent protein (GFP) into the ARC of transgenic TH-Cre mice and using fluorescent imaging to examine where in the brain the GFP-expressing axons project. Each reflection prompt below is accompanied by Judy's reflections as she develops her experimental design via the AiMS worksheet*.

### Phase 1: awareness

The first stage of the Three A's, Awareness, is about pausing to take stock of the experimental system. Too often, we rush ahead with familiar techniques without first considering what its key features are, what it can reveal, and whether the system is truly suited to the question at hand. Awareness provides the foundation for more informed choices in later stages of analysis and adaptation.

As a precondition to considering your experimental system, you must first define your research question. A well-defined research question serves as the foundation of experimental design, providing a clear anchor for the reflections that follow. Establishing this focus helps ensure that considerations about experimental design choices, assumptions, and possible outcomes remain tied to a central scientific aim. When formulating research questions, we encourage you to consult existing frameworks, such as patient/population, intervention, comparison, and outcome and feasible, interesting, novel, ethical, and relevant. For a summary of these and other frameworks for defining and refining research questions, see Covvey et al. (2024).

*Let* *Us Reflect: As you prepare to articulate your experimental design, take a moment to identify the specific research question you want to solve*.

Judy defined her research question as: To which regions of the brain do ARC–TH neurons project?

#### Step 1 of AiMS worksheet: identity components of your experimental system

The first step to applying the AiMS framework is to identify core components of the experimental system. Explicitly outlining the Three M's—Models, Methods, and Measurements—at play brings into view elements that will shape both what you can learn with your experimental system and how you will interpret your results. Articulating these components provides a foundation for more structured reflection in later steps.

*Let* *Us Reflect: To address your research question, what Models, Methods, and Measurements do you plan to, or could you, use?*

Judy identified the following components of her experimental system:

1. Model: Transgenic TH-Cre mouse
2. Method: Cre-dependent AAV-GFP viral injection into the ARC of TH-Cre mice
3. Measurement: Detection of GFP+ fibers in brain sections

#### Step 2 of AiMS worksheet: examine features of your experimental system

After defining the basic components of the experimental system, the next step is to consider their functional properties. Examining Specificity, Sensitivity, and Stability highlights our underlying assumption and reveals where the system may be particularly robust or vulnerable. This exercise is not intended to yield precise quantification but to surface practical parameters that may influence how we obtain and interpret evidence.

*Let Us Reflect: Select one Model, Method, and Measurement that you articulated in Step 1. For each component, describe experimental parameters that map onto Specificity, Sensitivity, and Stability*.

Judy's reflections about her experimental system are given in Table 1.

**Table 1.** Example reflections for neuroanatomy case study, Step 2 of AiMS framework

Components of experimental system	Specificity	Sensitivity	Stability
Model: transgenic TH-Cre mouse	What is the expression pattern of the Cre transgene?	What is the expression level of the Cre transgene?	What is the stability of the transgene over generations? What is the consistency of transgene expression across cohorts, ages, and infected cells?
Method: Cre-dependent AAV-GFP viral injection into the ARC of TH-Cre mice	What is the spread of the viral injection? Which cell types are infected by the specific viral serotype?	What is the titer of the virus and the effective viral load delivered to the tissue? What is the extent of the viral infection?	What is the viability of the virus at the time of injection? Are virus titers consistent across batches? Is viral gene expression consistent across animals?
Measurement: detection of GFP+ fibers in brain sections	Which microscope filters and imaging settings do I need to detect my specific fluorescent signal? Does my imaging modality allow me to distinguish axon terminals from fibers of passage?	What is the detection limit for fluorescently labeled axons? What is the minimum fiber density needed to confidently identify a projection?	What is the integrity of the tissue and fluorescent signal at the time of imaging? To what extent are imaging and image processing settings standardized and consistent?

This table summarizes Judy's reflections on her experimental system, identifying experimental parameters that map onto Specificity, Sensitivity, and Stability for her experimental Model, Method, and Measurement.

The Awareness phase of the AiMS framework emphasizes making deliberate choices in selecting an experimental system. Explicitly identifying its components and considering their key properties can help establish a clearer foundation for design choices and avoid rushing ahead with unexamined assumptions. This groundwork provides the opportunity to ensure alignment of the general approach to the central research question and paves the way for deeper analysis in the next phases of the AiMS cycle.

### Phase 2: analysis

With an awareness of the key features of an experimental system, the next step is Analysis. This phase focuses on probing deeper: envisioning possible outcomes, considering how those outcomes might be interpreted, and recognizing where the system might fail. Thus, the Analysis phase helps us think critically about what the experimental system can and cannot tell us.

#### Step 3 of AiMS worksheet: consider multiple possible experimental outcomes

A useful starting point for analysis is to imagine the full range of possible outcomes an experiment could produce. Rather than focusing only on the anticipated or desired result, it is useful to consider what the experiment would look like if the hypothesis is not supported or if something unexpected occurs. This reflection exercise helps guard against common cognitive biases, such as confirmation bias—the tendency to value results that align with a preferred hypothesis while discounting contradictory evidence (Born, 2024). Shifting away from expecting a single “correct” result productively reframes every outcome, including null or unexpected findings, as potentially informative.

*Let* *Us Reflect: For at least one of your Measurements, describe multiple possible experimental outcomes you might observe. You do not yet need to describe how you might interpret each outcome*.

For her neuroanatomical tracing experiment, Judy has identified multiple possible outcomes for her measurement, detection of GFP+ fibers in brain sections:

1. Possible observation #1: Presence of GFP+ fibers in the ME only
2. Possible observation #2: Presence of GFP+ fibers in the ME and the PVH
3. Possible observation #3: Presence of GFP+ fibers in the ME and PVH, as well as additional brain regions
4. Possible observation #4: Presence of GFP+ fibers in the ME and additional brain regions, but not the PVH

#### Step 4 of AiMS worksheet: distinguish biological interpretation versus technical artifact

A given experimental outcome is rarely self-explanatory. The same observed outcome could arise from valid biology or from technical artifacts/limitations of the system. A strong signal in the expected direction, for instance, might genuinely reflect the underlying biological process, but it could also arise from off-target effects, cross-reactivity, or unintended variation in the system. Similarly, the absence of a signal might indicate that the hypothesized effect does not exist, or it may simply reflect insufficient sensitivity, inadequate sample quality, or measurement error. By explicitly considering both biological explanation and technical artifact interpretation for any results, we can strengthen our ability to design appropriate controls, interpret data cautiously, and avoid premature or overconfident conclusions.

*Let* *Us Reflect: For each of the possible observed outcomes that you identified, describe at least one biological interpretation of the observation and at least one technical artifact interpretation*.

Upon reflection, Judy realized that the possible experimental outcomes she listed might have different interpretations. Her reflections capturing potential biological interpretations versus technical artifact interpretations are given in Table 2.

**Table 2.** Example reflections for neuroanatomy case study, Step 4 of AiMS framework

Possible observation	Biological interpretation	Technical artifact interpretation (which needs to be ruled out through experimental design)
Presence of GFP+ fibers in the ME but nowhere else	ARC–TH neurons project just to the known target (ME), nowhere else. This would be consistent with prior research but inconsistent with Judy's working hypothesis	Lack of fibers in other regions (including where the projection may only be minor) could be observed if there was poor infection of ARC–TH neurons or if infected neurons only weakly expressed GFP
GFP+ fibers observed in ME as well as in PVH	ARC–TH neurons project both to the known target (ME) and to a novel target, PVH. This would be consistent with Judy's hypothesis	The presence of fibers in the PVH—without ARC–TH neurons actually projecting there—could also be observed if the virus infected non-TH+ cells (e.g., due to leaky Cre) or if the viral infection extended beyond the ARC to infect other cells
The presence of GFP+ fibers in the ME and PVH, as well as additional brain regions	ARC–TH neurons project both to the known target (ME) and to a set of novel targets, including the PVH and other regions. This would be consistent with Judy's hypothesis but also gives an unexpected result	(Same possible artifacts described in the rows above that could yield either false-positive or false-negative anatomical results)
The presence of GFP+ fibers in the ME and additional brain regions, but not the PVH	ARC–TH neurons project both to the known target (ME) and to a set of novel targets that do not include the PVH. This would be inconsistent with Judy's hypothesis	(Same possible artifacts described in the rows above that could yield either false-positive or false-negative anatomical results)

This table summarizes Judy's reflections on possible biological interpretations and possible technical artifact interpretations for different possible experimental outcomes.

#### Step 5 of AiMS worksheet: identify potential experimental failure points

Now that we have explored ways that technical artifacts might confound our interpretation of experimental outcomes, let us explore further how the experimental system itself can break down. Weaknesses can arise at the level of the Model, such as when it does not fully capture the phenomenon of interest; at the level of the Method, such as when the intervention is applied inconsistently or with limited efficiency; or at the level of the Measurement, such as when experimental readouts are distorted by noise or signal loss over time. Thinking through these weak points does not assume that experimental failure is inevitable, but it highlights where evidence could be compromised. By acknowledging these risks upfront, we can build appropriate data collection into our experimental design to monitor our experimental system, as discussed in the next section, rather than discovering the problems only after data collection, thereby strengthening the reliability of our results.

*Let* *Us Reflect: Identify ways in which the Model, Method, and Measurement components of your experimental system could potentially fail along the dimensions of Specificity, Sensitivity, or Stability*.

Judy noted that her system has certain vulnerabilities. Some of her ideas for how her system could fail are depicted in Table 3.

**Table 3.** Example reflections for neuroanatomy case study, Step 5 of AiMS framework

Components of Experimental System	Specificity - potential ways it could break/fail	Sensitivity - potential ways it could break/fail	Stability - potential ways it could break/fail
Model: transgenic TH-Cre mouse	Transgenic Cre expression may not faithfully reflect endogenous TH expression	Cre expression and/or activity may be insufficient to induce GFP expression in target cells	Cre expression may change over time due to genetic drift in mouse lines (or mis-genotyping) Cre expression may be variable across mice and litters Cre expression may vary with age/developmental stage of mice and/or may be heterogeneous among infected cells
Method: Cre-dependent AAV-GFP viral injection into the ARC of TH-Cre mice	AAV injection might not be limited to the target brain region (ARC) Viral serotype may preferentially infect non-neuronal cells Since the GFP fills the entire cell, we may not be able to easily distinguish fibers of passage from axon terminals	Virus volume, titer, or promoter activity may be insufficient for GFP expression upon Cre recombination within cells Virus may only infect a small proportion of ARC–TH+ cells	Virus viability may have been lost due to poor handling (e.g., kept at room temp) Viral titers and expression levels may vary by batch and across animals Injection volumes and/or coordinates may drift over time due to equipment issues
Measurement: detection of GFP+ fibers in brain sections	May be difficult to determine sites of axon termination in brain sections May be imaging artifacts (e.g., autofluorescence, bleed through) due to imaging and/or filter settings	Fluorescent signal may be too low for microscope to detect (i.e., below limit of detection) Fiber density may be too sparse to confidently identify a projection	Tissue integrity and fluorescent stability (e.g.*,* photobleaching) may degrade Imaging and image processing settings may not be standardized or consistent across samples

This table summarizes Judy's reflections on how the Model, Method, and Measurement components of her experimental system could potentially fail along the dimensions of Specificity, Sensitivity, or Stability.

Taken together, these reflections highlight that the Analysis phase is not about predicting a single correct answer. It is about mapping the terrain of possible outcomes, alternative explanations, and failure points. This process is not intended to eliminate uncertainty but make it visible and manageable, paving the way for the final phase of our metacognitive cycle: Adaptation.

### Phase 3: adaptation

Having identified key features of an experimental system (Awareness) and anticipated its possible outcomes and failure modes (Analysis), we now transition to the final stage of the Three A's: Adaptation. Adaptation is about acting on reflection: introducing strategies that strengthen interpretation, recognizing when technical limitations may be insurmountable, and making deliberate choices about how to proceed.

#### Step 6 of AiMS worksheet: plan to monitor performance of your experimental system

As discussed previously, a recurring challenge in experimental design is distinguishing whether a result reflects true biology or technical artifact. We can reduce this uncertainty by “collecting data”—through the design of experimental controls—on how the experimental system itself behaves during the experiment. “Collecting data” to probe the Specificity, Sensitivity, and Stability of your experimental system—such as by validating reagents, determining dynamic range, or assessing replicability across contexts—can further clarify how well the experimental system is performing. These steps do not eliminate uncertainty, but they increase confidence that results are interpretable in biological terms rather than reflecting system artifacts.

*Let* *Us Reflect: Select a subset of potential technical failure modes you identified during Step 5. Brainstorm ways you could “collect data” on whether the technical failure might have occurred when you run your experiment*.

Judy reflected on how to monitor the performance of her experimental system. Her description of some of the potential technical failures she might encounter and ways to “collect data” to monitor whether these failures might have occurred are summarized in Table 4.

**Table 4.** Example reflections for neuroanatomy case study, Step 6 of AiMS framework

Potential technical failure	Way(s) to “collect data” as to whether the experiment actually failed in this way
Cre expression may not faithfully reflect TH expression	Perform double IHC for Cre and endogenous TH and evaluate overlap
May be insufficient virus injected for GFP expression upon Cre recombination within cells (or insufficient activity of promoter)	Analyze sections for presence of GFP in cell bodies; repeat using viral titrations of different volumes, dilutions and/or different time courses for GFP expression and trafficking before sectioning the brain
Tissue integrity and/or degraded fluorescent signal may impair imaging quality	Perform additional histological analysis of the tissue to assess integrity; adjust imaging settings between different brain sections from the same animal to compare imaging quality (in case photobleaching was an issue); calibrate imaging conditions according to a standard (e.g., prepared slide of fluorescent pollen)

This table summarizes Judy's reflections on ways she could “collect data” on whether various potential technical failures of her experimental system might have occurred when she runs her experiment.

We have structured our discussion here to encourage broad thinking about how you could monitor the performance of your experimental system without imposing constraints of precise labeling or classification of specific experimental control conditions. However, such classification can be helpful as you continue to build out your experimental approach. We encourage you to explore some of the many excellent educational resources that elaborate upon different types of experimental controls (Glass, 2014; Harrington, 2020; Community for Rigor, n.d.) and to incorporate relevant concepts into your fully articulated experimental plan.

#### Step 7 of AiMS worksheet: identify potential experimental pitfalls and alternative experimental approaches

All experimental systems have their technical limitations, and some technical limitations cannot be addressed or overcome through experimental controls that provide additional data on the experimental system. These potential experimental pitfalls represent possibly insurmountable technical constraints that would prevent a system from addressing the research question. Reflecting upon potential pitfalls can allow us to avoid investing resources in a design that cannot yield interpretable answers. In some cases, this may mean redefining the research question; in others, it may involve a shift to an alternative experimental approach with a different Model, Method, or Measurement that enables you to bypass technical limitations of your initial experimental system.

*Let* *Us Reflect: Which of the technical limitations that you identified for your experimental system are most likely to pose technical pitfalls that are not easy to overcome through troubleshooting? Also consider other aspects of your experimental system not captured in the previous reflection exercises that may also present potential experimental pitfalls (e.g., inherently challenging experiments to execute, untested approaches). What alternative approaches could enable you to overcome the potential experimental pitfalls that you identified? Try to identify at least one alternative model, method, and measurement*.

Judy recognized several potential experimental pitfalls of her system and began considering alternative experimental approaches to answer her research question. A sampling of her ideas is presented in Table 5.

**Table 5.** Example reflections for neuroanatomy case study, Step 7 of AiMS framework

Components of Experimental System	Potential Experimental Pitfall (can't overcome)	Alternative Experimental Approach
Model: transgenic TH-Cre mouse	If the Cre expression is not entirely restricted to TH+ neurons, this will make interpretation of the results challenging or impossible, since non-TH+ cells in the ARC have been shown to project to the PVH	Explore whether there is a targeted knock-in mouse available or opportunities for an intersectional approach (Cre/FLP)
Method: Cre-dependent AAV-GFP viral injection into the ARC of TH-Cre mice	It may not be possible to infect sufficient numbers of ARC–TH neurons while confining viral injection within the ARC, yielding incomplete/weak projection patterns	Could complement this approach with retro AAV (rAAV) tracing, which may enable larger injection volume in different brain regions in a candidate-based approach because it does not require selective labeling of projections from ARC, and then assess colocalization of rAAV-fluorophore and TH in the ARC cells. Could also use bulk injection of retrogradely transported dyes as a first-pass
Measurement: detection of GFP+ fibers in brain sections	It may be challenging to differentiate fibers of passage from synaptic terminals by imaging brain sections	Could attempt volumetric imaging using a tissue-clearing approach or use imaging software that can reconstruct volumes from tissue sections. Alternatively, could colabel neurons with a synaptic marker like synaptophysin (e.g., virally codelivered with the fluorescent tracer in a Cre-dependent manner)

This table summarizes Judy's reflections on potential experimental pitfalls for her current experimental system and alternative experimental approaches to answer her research question.

#### Step 8 of AiMS worksheet: apply a critical lens to your initial and alternative experimental approaches

Notably, exploring alternative approaches does not always mean replacing one approach with another. Complementary strategies can strengthen findings by corroborating results across methods or adding new layers of evidence. In neuroscience research, for example, optogenetic and chemogenetic approaches are often paired (Krut’ et al., 2024; Meron Asher and Goshen, 2025; S. Li et al., 2025); viral neuroanatomical tracing can be combined with electrophysiology (Kim et al., 2017), and calcium imaging is interpreted alongside electrophysiology recordings (Wei et al., 2020). Emerging technologies like spatial transcriptomics illustrate how integrating distinct methods can leverage the relative advantages of both methods, underscoring the value of complementary techniques (Williams et al., 2022). Considering such complementary approaches at the design stage allows us to plan for both corroboration and elaboration, increasing confidence in our conclusions.

At the same time, reflecting on alternative experimental approaches provides an opportunity to critically examine the factors that drove our initial selection of the experimental system. Such factors can include both scientific rationale (e.g., perhaps your method offers the greatest sensitivity) and legitimate logistical reasons (e.g., perhaps the mouse model you are using was already available at your institution), but it might also be influenced by cognitive biases (Do you perceive a particular experimental method or analysis approach to be “superior” just because it has been extensively used in your laboratory? Have you taken the time to reflect on whether it is actually the best-suited approach?). Thus, we provide a final prompt to structure reflection on your initial experimental system to further mitigate the influence of cognitive biases and increase the rigor of your approach.

*Let* *Us Reflect: What were the primary factors driving your selection of your initial experimental Models, Methods, and Measurements? What factors led you to not pursue your proposed “alternative” approaches in your initial experimental design? Are there any advantages to trying your “alternatives” from the outset or in parallel to your initial experimental design as a complementary approach?*

Judy generated many insights about her initial experimental design decisions, some of which are presented below:

1. She deemed the specific viral tracing strategy using a Cre-dependent virus injected into the ARC of TH-Cre mice to be the best approach to selectively express the GFP tracer in the dopaminergic cells of the ARC to characterize their axonal projections in a specific, yet unbiased, fashion. If she had crossed the TH-Cre mouse to a Cre-dependent GFP reporter line, she would have lost the spatial specificity and would have indiscriminately labeled all TH+ neurons, including those in other parts of the hypothalamus. She selected the specific TH-Cre transgenic mouse line based on its availability in a commercial mouse repository and because it had been previously characterized in published research. She initially chose to measure the presence of GFP+ fibers in brain sections because it seemed sufficient to answer her research question; a large body of prior neuroanatomical studies had characterized axonal projection patterns via fiber detection in brain slices.
2. Judy did not initially pursue whole-mount brain imaging to characterize the pattern of ARC–TH axonal projections not only because of the precedent that imaging of brain sections would be sufficient but also because it would require the development of novel brain clearing and imaging protocols in her lab and access to a light sheet microscope, which was not readily available in her department.
3. Judy described using a retroAAV viral anatomical tracing approach—in which she would inject a Cre-dependent retroAAV at the predicted sites of ARC–TH axonal terminals to retrogradely label their cell bodies—as a potential alternative approach to her anterograde tracing strategy of injecting ARC–TH neuron cell bodies to label their axons. As a primary strategy, this approach has the limitation of being candidate-based, as opposed to unbiased, only returning information about whether ARC–TH neurons project to the specific regions where the virus was introduced. However, this would be a useful approach to validate potential target regions identified from the anterograde tracing experiments or could even be used in parallel with the anterograde tracing strategy to test Judy's specific hypothesis that ARC–TH neurons project to the PVH.

Thus, we complete a full revolution of our AiMS metacognitive cycle. Through the Adaptation phase, you make explicit decisions based on what has been learned through Awareness and Analysis while also identifying alternative experimental approaches. This, in turn, should trigger a return to the Awareness and Analysis phases for further reflection to refine your experimental design before such alternative experimental approaches are implemented. Importantly, the iterative nature of this metacognitive process is meant to guide, not paralyze, you. Experimental design often involves committing to a design despite known limitations, provided those limitations are acknowledged and managed. It may also involve pivoting to alternative strategies or layering complementary approaches to strengthen conclusions. What matters is not that every design is perfect—few, if any, ever are—but that choices are made deliberately, with a clear-eyed view of the assumptions, vulnerabilities, and trade-offs involved.

## From Structured Thinking to Structured Writing: Application to Research Proposals

Fellowship and grant proposals provide one of the primary venues where experimental design is formally articulated. These documents emphasize brevity, typically imposing strict word or page limits that preclude a comprehensive description of every experimental design choice, assumption, and possible alternative. Nevertheless, completing the type of structured reflection outlined in the AiMS framework remains valuable. Even if the full details of your responses cannot be included in a written research proposal, these reflections can help clarify the logic of the experiment and make it easier for you to distill the most critical points for reviewers.

In our experience teaching graduate-level research proposal writing courses, we have observed recurring misconceptions that the AiMS framework is designed to counteract. An often-encountered example is framing experiments as binary verdicts—“If I observe [the predicted outcome that aligns with my hypothesis], I will conclude [the biological interpretation]. Otherwise, I will conclude that *my experiment has failed*.” Closely related, students sometimes define “pitfalls” as the absence of their predicted outcome—“a potential pitfall is that *I won't observe the outcome that is aligned to my hypothesis*.” This flawed reasoning overlooks the possibility that the same observation could reflect either an underlying biological reality or a technical artifact of the system and that unexpected or null results can still be informative if properly controlled for and interpreted. The AiMS framework—through guided reflections on anticipating multiple outcomes, distinguishing biological interpretation from technical artifacts, and identifying genuine pitfalls—was designed to mitigate these misconceptions, thereby supporting more rigorous reasoning in research proposals.

Indeed, information from the completed AiMS worksheet maps readily onto the structure of research proposals. Proposal formats commonly require a clear statement of the research question, description of the experimental system, discussion of experimental controls, identification of multiple possible experimental outcomes and their interpretations, and consideration of potential pitfalls and alternative experimental approaches. Each of these aligns directly with reflection prompts in our AiMS framework. Thus, engaging with the worksheet before drafting a proposal can help streamline the writing process: the reflective work provides a reservoir of ideas and reasoning that can be selectively condensed into the research proposal format while ensuring that the proposal conveys to reviewers that the experiment has been carefully thought through in terms of both strengths and possible limitations. As we describe in the following section, generative AI tools can also play a role in this process, serving as a metacognitive partner that helps organize reflections from the AiMS framework into proposal-ready formats while preserving the original contribution of the researcher.

## Generative AI as a Metacognitive Partner

Generative AI (Gen AI) tools, such as ChatGPT, present both opportunities and challenges as they become integrated into the scientific discovery process (Wang et al., 2023; Kwon, 2025; Naddaf, 2025). The current Gen AI landscape is evolving quickly. Multiple funding agencies, publishers, and institutions have recently issued guidance on the appropriate use of Gen AI, emphasizing transparency and disclosure. For example, the National Institutes of Health (NIH) clarified in a July 2025 Notice (National Institutes of Health, 2025) that grant proposals must reflect the original intellectual contribution of the applicant(s) and should not be “substantially developed by AI.” Multiple journals, including *eNeuro* and its parent Society for Neuroscience, have adopted the International Committee of Medical Journal Editors recommendation that AI-assisted writing must be disclosed (ICMJE, n.d.). Ethical concerns further complicate the use of Gen AI tools, including known biases inherent in large language models (LLMs), legal questions of copyright and ownership, risks to data privacy when sharing sensitive or unpublished materials, and the significant environmental footprint of these tools (Weidinger et al., 2021; Masters, 2023; Tran et al., 2025). Taken together, these developments reflect a new normal in scientific research: Gen AI tools should be used to “enhance,” but not “replace,” the original contributions of researchers and must be engaged responsibly and transparently.

Within this context, both our explorations and recent work demonstrating the strong metacognitive capabilities of Gen AI LLMs (Pavlovic et al., 2024) suggest that Gen AI holds promise for enhancing the rigor of experimental design when used as a “metacognitive partner,” not by producing answers but by encouraging deeper and more structured reflection. For example, when we prompted ChatGPT with “I am going to describe my experimental design for a neuroscience experiment. Act as a metacognitive partner who prompts me with questions to probe aspects of my design to enhance the experimental rigor and combat possible cognitive biases embedded within the design,” its output included thoughtful questions spanning multiple aspects of the experimental design (see Fig. 2 for an excerpt of the interaction with ChatGPT). This interaction demonstrates how Gen AI can be recruited to strengthen reflective practices and make the experimental design process more deliberate.

Building on this example, we found that Gen AI can take on several roles that enhance experimental design using the AiMS metacognitive framework: as a brainstorming partner, a constructive reviewer, or an organizing assistant. Each of these roles is described in general terms below; specific prompts for use with Gen AI that reflect each of these roles, as well as prompts to critically engage with your Gen AI partner, are given in Box 1. As a brainstorming partner, Gen AI can scaffold the reflection process through structured prompts that surface assumptions and clarify reasoning. You can also prompt it to expand on initial ideas, propose alternatives, and generate suggestions under specific constraints. In this role, Gen AI does not replace original thinking but can help deepen and broaden reflections, encouraging considerations beyond your first instincts. As a constructive reviewer, Gen AI can be asked to critique an experimental plan from the perspective of a grant reviewer, journal referee, or a general critic. It can point to missing controls, highlight how confirmation bias might influence interpretation, or flag other potential weaknesses. In some cases, it can help prioritize which vulnerabilities are most critical to address. It can even be prompted to critique its own outputs, a meta-exercise that can improve the quality or breadth of the responses. Used this way, Gen AI functions not as an idea generator but as a critic that encourages you to evaluate your design from the standpoint of rigorous peer review. As an organizing assistant, Gen AI can help map content from a completed AiMS worksheet into other formats, such as experimental proposals or manuscript methods sections. This may include aligning your reflective responses with reporting guidelines such as the ARRIVE guidelines (Percie du Sert et al., 2020) or the Cell Press STAR Methods (Marcus, 2016). By organizing and reformatting information, GAI can make the underlying logic of your experimental design clearer to others while still preserving your original intellectual contribution.

Box 1. Example prompts for using generative AI in experimental design. Generative AI can adopt different roles and support experimental design by facilitating metacognitive reflections. Below are example prompts that align with the roles of a brainstorming partner, a constructive reviewer, and an organizing assistant, as well as ones that prompt Gen AI to appraise its own output. These prompts have been tested with a combination of ChatGPT models 4o and 5. These prompts can be adapted to individual research contexts or serve as inspiration for new prompts.

**The brainstorming partner**

*Goals: broaden ideas and surface overlooked considerations*

- “I am going to describe my experimental design for a neuroscience experiment. Act as a metacognitive partner who prompts me with questions to probe aspects of my design to enhance the experimental rigor and combat possible cognitive biases embedded within the design.”
- “Using structured prompts and questions, guide me to complete the attached worksheet for my specific experimental system. You should provide guiding questions but do not actually complete the table. This is meant to be an exercise to guide my thinking, but not to provide me with the answers.” [Upload blank AiMS worksheet]
- “Review the attached experimental plan. Using your expertise as a neuroscience researcher, elaborate on each component of this worksheet and regenerate an expanded version with additional examples provided for each prompt.” [Upload completed AiMS worksheet].
- “For my model, method, and measurement, can you identify additional examples of specificity, sensitivity, and stability?” [Upload completed table for reflection prompt #2.]
- “I am a researcher who wants to investigate a similar research question as that described above (where in the rodent brain do ARC–TH neurons project) but my lab is resource-limited and we don't have the biosafety clearance to perform viral neuroanatomical tracing experiments. Propose equally rigorous experimental approaches that I could use instead.”
- “I am a graduate researcher writing my first research proposal. What are some common misconceptions held, or errors made, by research trainees related to (1) identifying multiple possible outcomes to their experiments, (2) identifying potential pitfalls to their experiments, and (3) proposing alternative approaches when writing their research proposals? Propose strategies that I can take to avoid making these common mistakes.”

**The constructive reviewer**

*Goals: probe weakness or biases and provide constructive feedback*

- “You are a leading neuroscience researcher and expert in principles of experimental rigor. Review the attached experimental plan and generate a series of guiding questions to be delivered as feedback to the researcher to highlight areas that need to be strengthened.” [Upload completed AiMS worksheet]
- *(To prompt GAI to evaluate its own output.)* “You are now a reviewer analyzing an experimental design that includes the experimental controls that you just suggested. Provide constructive feedback as to what is missing and what technical artifacts may still arise, despite the inclusion of these controls. Provide concrete suggestions for how the experimental plan could be strengthened according to published frameworks of rigorous experimental design. Provide real and accurate citations.”
- “Review my uploaded experimental plan. How might my brainstorming about possible experimental outcomes be influenced by confirmation bias or other cognitive biases?” [Upload completed AiMS worksheet]
- “Analyze the potential technical limitations or failure modes described in the attached experimental plan and prioritize them based on the likelihood of their occurrence. What aspects of experimental design should I prioritize to address the most likely technical issues?” [Upload completed AiMS worksheet]
- “You are a professor chairing a preliminary qualifying examination committee for a PhD student. Use the uploaded AiMS worksheet to generate probing questions about the experimental design to test the PhD student's knowledge of their experiment and principles of experimental rigor. Give one question at a time and then respond to my answer as if we are engaged in the questioning during the examination.” [Upload completed AiMS worksheet]

**The organizing assistant**

*Goals: organize ideas and reframe information*

- “You are a professor chairing a preliminary qualifying examination committee for a PhD student. Use the uploaded AiMS worksheet to generate probing questions about the experimental design to test the PhD student's knowledge of their experiment and principles of experimental rigor. Give one question at a time and then respond to my answer as if we are engaged in the questioning during the examination.” [Upload completed AiMS worksheet]
- “I'm attaching a worksheet that describes aspects of my experimental design. Map this information onto an outline for an NRSA-style NIH research proposal, noting which information I should prioritize for inclusion in the proposal given the restricted page limit and how I should organize it according to standard grant headings.” [Upload completed AiMS worksheet]
  ○ *Alternative*: “Re-do the prior analysis. Rather than directly mapping the content of the worksheet onto an NRSA-style NIH proposal outline, generate a series of guiding questions that prompt me to do so.”
- “Please map the information contained within the attached worksheet onto the ARRIVE guidelines (described here: https://arriveguidelines.org/). Highlight gaps in my experimental plan that I need to address to meet the ARRIVE guidelines for reporting.” [Upload completed AiMS worksheet]

**Critically engaging with Gen AI**

*Goals: prompt Gen AI to evaluate its own output and provide tools for human verification of AI*

- “Perform an honest and self-critical appraisal: what are the relevant strengths and limitations of generative AI tools like ChatGPT 5 to assist human scientists in experimental design?”
- “I'm using genAI to help create metacognitive worksheets to guide scientific trainees in experimental design. Identify the ways in which genAI might NOT be helpful toward this goal.”
  ○ Follow-up: “For each of the above limitations given, provide some metacognitive reflection questions that engage the learner in thinking to mitigate the limitation.”
- “Please attempt to validate each of the citations you provided above. If incorrect, please identify the correct citations.”
- “Generate a checklist for scientific trainees for how to vet the information that is generated from GenAI.”

**Figure 3.**
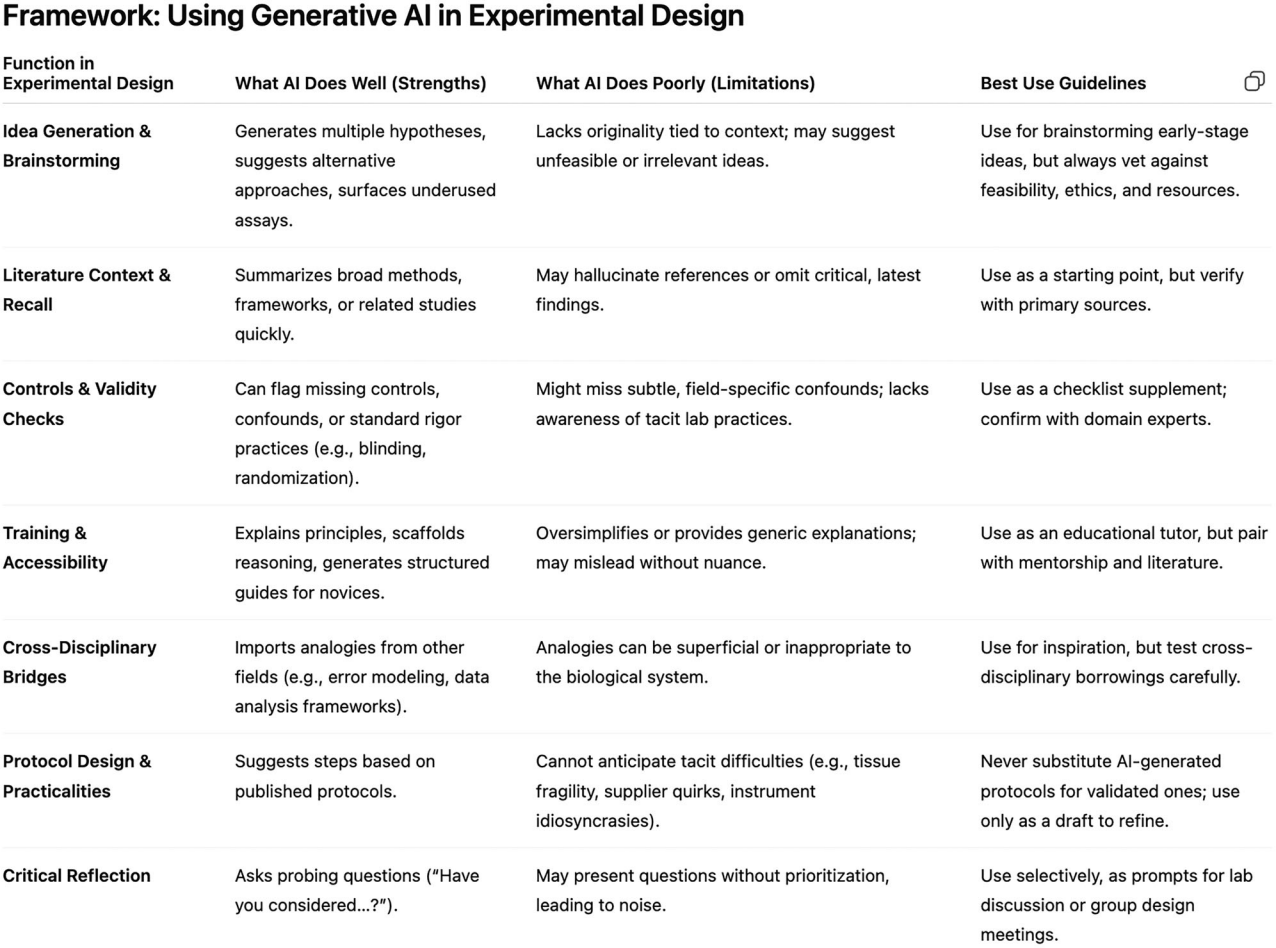
Strengths and limitations of using gen AI in experimental design, according to ChatGPT. A screenshot of the table generated by ChatGPT5 (university enterprise account) summarizes the output in response to the prompt: “Perform an honest and self-critical appraisal: what are the relevant strengths and limitations of generative AI tools like ChatGPT 5 to assist human scientists in experimental design?”.

Despite these potential benefits of using Gen AI in supporting experimental design, it also poses important limitations to keep in mind. In a meta-reflective exercise, we asked ChatGPT5 to perform a self-critical appraisal of its strengths and limitations in assisting researchers in experimental design. Its output (Fig. 3) echoes our own observations and aligns with known limitations of Gen AI, such as producing generic responses—which could result in an overall loss of creativity—plausible but inaccurate information and hallucinated citations (Weidinger et al., 2021; Huang et al., 2025; Peters and Chin-Yee, 2025). Ultimately, researchers are responsible for the accuracy and integrity of any information that Gen AI may supply. For this reason, careful verification, critical evaluation, and alignment with institutional and disciplinary policies are essential. Responsible use requires informed decisions that reflect both professional standards and values.

Our experience exploring the use of Gen AI in experimental design, consistent with others’ (Steinauer, 2025), suggests that Gen AI is most effective when used as a sparring partner rather than as an answer generator. Although the speed of Gen AI may tempt you to seek quick solutions, engaging with it might be most valuable when it slows you down, prompting you to question assumptions, examine alternatives, and strengthen your experimental design. In this way, using Gen AI as a metacognitive partner reinforces the central theme of the AiMS framework: structured reflection, not shortcuts, is what ultimately strengthens experimental design.

## Conclusion

This paper introduces the AiMS framework as a structured approach to experimental design rooted in metacognitive reflection. The Three A's—Awareness, Analysis, and Adaptation—provide a reflective cycle for recognizing the features of an experimental system, envisioning outcomes and limitations, and refining strategies in response. The Three M's—Models, Methods, and Measurements—help disentangle the moving parts of an experimental system, while the Three S's—Specificity, Sensitivity, and Stability—offer heuristics for evaluating their strengths and weaknesses. Together, these tools encourage researchers to move beyond procedural execution toward deliberate, reflective choices in experimental design that acknowledge vulnerabilities and trade-offs. We note that the AiMS framework does not capture many essential principles of rigorous experimentation, such as randomization and blinding, the use of replicates, and best practices for data analysis. These principles and practices, described in detail in many excellent resources (Glass, 2014; Harrington, 2020; Community for Rigor, n.d.; National Institute of General Medical Sciences, n.d.), remain indispensable. The AiMS framework is intended to complement, rather than replace, such standards by foregrounding structured reflection on experimental systems. While demonstrated here with a neuroanatomical case study, the AiMS framework is adaptable to other areas of neuroscience and other domains of life science research. We also explored how generative AI, when used responsibly as a metacognitive partner, can extend this reflective process without supplanting original intellectual contribution. Taken together, our goal is not to prescribe rigid rules but to provide tools that help researchers think more critically and creatively about their experimental design choices. Looking ahead, integrating structured reflection practices alongside established best practices and engaging responsibly with emerging technology can help foster rigor, creativity, and adaptability in the evolving landscape of scientific discovery.

## Generative AI Usage Statement

Generative AI (ChatGPT4o and 5, OpenAI) was used in testing the application of the AiMS framework and examining its potential to serve as a metacognitive partner in experimental design. Some of its outputs are included in the text as examples and are clearly indicated. During the manuscript writing process, ChatGPT5 was occasionally used for text editing. All content of this manuscript has been critically reviewed, revised, and approved by the authors, who take full responsibility for the accuracy and integrity of the manuscript.

## Synthesis

Reviewing Editor: Nathalie Ginovart, University of Geneva Decisions are customarily a result of the Reviewing Editor and the peer reviewers coming together and discussing their recommendations until a consensus is reached. When revisions are invited, a fact-based synthesis statement explaining their decision and outlining what is needed to prepare a revision will be listed below. The following reviewer(s) agreed to reveal their identity: Robert Calin-Jageman.

Review of yor paper is complete, and eNeuro is enthusiastic about your contribution to the "Improving Your Neuroscience" series. I have copied the review below which contains 2 minor revisions to consider.

This paper describes a educational framework for fostering strong experimental design in neuroscience. That is, it provides a set of ideas, a worksheet, and a guided case study that neuroscientists can utilize to help improve their own thinking about study design and/or adopt as part of their training curriculum. The framework prompts to go through three stages of reflection (Awareness, Analysis, Adaptation), during which they make explicit their Models, Methods, and Measurements, and consider for each their Specificity, Sensitivity, and Stability. From just listing the steps/themes I risk making this sound simplistic - the actual paper and case study make clear it is not! The case study is particularly compelling in showing how taking the time to just carefully describe and reflect on a future study in a structured way can really open up the researcher to considering important controls, the potential for integrating mixed methods, and more. The researchers make an especially compelling point that working through this process would also help with grant writing! They also have an intriguing section on how a researcher might use an LLM as a "sparring partner" to help them with their design process - this section is not essential to the paper, but I suspect most eNeuro readers will find the thoughts here very intriguing, providing some very useful thoughts, prompts, and references for how LLMs might actually prove useful in this context.

Overall, I am very enthusiastic about this contribution. The paper is well-written, clearly organized, and it the case study really demonstrates the value of this type of structured reflection during experiment planning. The materials needed to adopt this framework are provided as extended figures.

I have only a few suggestions for the authors:

• First, the authors indicate in their submission comments that these materials were developed in part through an NIH grant to produce training materials for rigor and reproducibility and that they will eventually become part of a publicly-available training module. That module is not yet online, but I think it is worth a paragraph before the case study giving the reader this important context about how/why this framework was developed, and perhaps a pointer to how they would eventually be able to find these materials as adoptable training modules.

• Page 4, line 97 - the paper is written in an inviting style, but I think here the authors may want to amend just a bit: first, because I don't think this paper is only for early-career researchers, and second because the "you" here seems a bit out of sorts for *this* format (journal publication) (though it is perfectly suitable as part of a training module)

## Author Response

September 28, 2025 We thank the editors for their positive feedback on our manuscript. We have addressed each of the two comments that were provided, as indicated below: • First, the authors indicate in their submission comments that these materials were developed in part through an NIH grant to produce training materials for rigor and reproducibility and that they will eventually become part of a publicly-available training module. That module is not yet online, but I think it is worth a paragraph before the case study giving the reader this important context about how/why this framework was developed, and perhaps a pointer to how they would eventually be able to find these materials as adoptable training modules.

- We have added a paragraph immediately preceding the interactive tutorial section of the article explaining the relationship to the C4R initiative. • Page 4, line 97 - the paper is written in an inviting style, but I think here the authors may want to amend just a bit: first, because I don't think this paper is only for early-career researchers, and second because the "you" here seems a bit out of sorts for *this* format (journal publication) (though it is perfectly suitable as part of a training module) - We have amended the language to make it more formal and to refer to "researchers," not just early-career researchers.

We thank you for your support of our work.

Taralyn Tan and Jade Li

## References

[B1] Born RT (2024) Stop fooling yourself! (diagnosing and treating confirmation bias). eNeuro 11:ENEURO.0415-24.2024. 10.1523/ENEURO.0415-24.2024 PMC11495861 39438140

[B2] Community for Rigor (n.d.) Units [WWW Document]. https://www.c4r.io/units (accessed 8.31.25).

[B3] Covvey JR, McClendon C, Gionfriddo MR (2024) Back to the basics: guidance for formulating good research questions. Res Social Adm Pharm 20:66–69. 10.1016/j.sapharm.2023.09.009 37838572 PMC11129835

[B4] Glass DJ (2014) *Experimental design for biologists*, Ed 2. Cold Spring Harbor (N.Y.): Cold Spring Harbor Laboratory Press.

[B5] Harrington ME (2020) *The design of experiments in neuroscience*, Ed 3. Cambridge: Cambridge University Press.

[B6] Holder DJ, Marino MJ (2017) Logical experimental design and execution in the biomedical sciences. Curr Protoc Pharmacol 76:A.3G.1–A.3G.26. 10.1002/cpph.20 28306153

[B7] Huang L, et al. (2025) A survey on hallucination in large language models: principles, taxonomy, challenges, and open questions. ACM Trans Inf Syst 43:1–55. 10.1145/3703155

[B8] ICMJE (n.d.) Recommendations | Defining the role of authors and contributors [WWW Document]. https://www.icmje.org/recommendations/browse/roles-and-responsibilities/defining-the-role-of-authors-and-contributors.html (accessed 8.31.25).

[B9] Kim CK, Adhikari A, Deisseroth K (2017) Integration of optogenetics with complementary methodologies in systems neuroscience. Nat Rev Neurosci 18:222–235. 10.1038/nrn.2017.15 28303019 PMC5708544

[B10] Krut’ VG, Kalinichenko AL, Maltsev DI, Jappy D, Shevchenko EK, Podgorny OV, Belousov VV (2024) Optogenetic and chemogenetic approaches for modeling neurological disorders in vivo. Prog Neurobiol 235:102600. 10.1016/j.pneurobio.2024.102600 38548126

[B11] Kwon D (2025) Is it OK for AI to write science papers? Nature survey shows researchers are split. Nature 641:574–578. 10.1038/d41586-025-01463-8 40369145

[B12] Li S, Zhang J, Li J, Hu Y, Zhang M, Wang H (2025) Optogenetics and chemogenetics: key tools for modulating neural circuits in rodent models of depression. Front Neural Circuits 19:1516839. 10.3389/fncir.2025.1516839 40070557 PMC11893610

[B13] Li X, Patrnogić J, Van Vactor D (2025) Flexible competency framework: a tool for optimizing life science training. PLoS Biol 23:e3003331. 10.1371/journal.pbio.3003331 40864622 PMC12385351

[B14] Marcus E (2016) A STAR is born. Cell 166:1059–1060. 10.1016/j.cell.2016.08.021 27565332

[B15] Masca NG, et al. (2015) RIPOSTE: a framework for improving the design and analysis of laboratory-based research. Elife 4:e05519. 10.7554/eLife.05519 25951517 PMC4461852

[B16] Masters K (2023) Ethical use of artificial intelligence in health professions education: AMEE guide no. 158. Med Teach 45:574–584. 10.1080/0142159X.2023.2186203 36912253

[B17] McCabe TM, Olimpo JT (2020) Advancing metacognitive practices in experimental design: a suite of worksheet-based activities to promote reflection and discourse in laboratory contexts. J Microbiol Biol Educ 21:130. 10.1128/jmbe.v21i1.2009 PMC7198226 32431775

[B18] Meron Asher S, Goshen I (2025) Chemogenetic and optogenetic tools revolutionizing the study of astrocytes in memory. Curr Opin Neurobiol 93:103057. 10.1016/j.conb.2025.103057 40440756

[B19] Naddaf M (2025) How are researchers using AI? Survey reveals pros and cons for science. Nature. 10.1038/d41586-025-00343-5 39905251

[B20] National Academies of Sciences, Engineering, and Medicine (2018) *Graduate STEM education for the 21st century*. Washington, D.C.: National Academies Press.

[B21] National Institute of General Medical Sciences (n.d.) Clearinghouse for modules to enhance biomedical research workforce training [WWW Document]. https://www.nigms.nih.gov/training/Pages/clearinghouse-for-training-modules-to-enhance-data-reproducibility (accessed 8.31.25).

[B22] National Institutes of Health (2025) NOT-OD-25-132: supporting fairness and originality in NIH research applications [WWW Document]. https://grants.nih.gov/grants/guide/notice-files/NOT-OD-25-132.html (accessed 8.31.25).

[B23] Pavlovic J, Krstic J, Mitrovic L, Babic D, Milosavljevic A, Nikolic M, Karaklic T, Mitrovic T (2024) Generative AI as a metacognitive agent: a comparative mixed-method study with human participants on ICF-mimicking exam performance. arXiv preprint arXiv:2405.05285.

[B24] Percie Du Sert N, et al. (2020) The ARRIVE guidelines 2.0: updated guidelines for reporting animal research. PLoS Biol 18:e3000410. 10.1371/journal.pbio.3000410 32663219 PMC7360023

[B25] Peters U, Chin-Yee B (2025) Generalization bias in large language model summarization of scientific research. R Soc Open Sci 12:241776. 10.1098/rsos.241776 40309181 PMC12042776

[B26] Schraw G, Moshman D (1995) Metacognitive theories. Educ Psychol Rev 7:351–371. 10.1007/BF02212307

[B27] Stanton JD, Sebesta AJ, Dunlosky J (2021) Fostering metacognition to support student learning and performance. CBE Life Sci Educ 20:fe3. 10.1187/cbe.20-12-0289 33797282 PMC8734377

[B28] Steinauer A (2025) My AI chatbot thinks my idea is fundable. Nature. 10.1038/d41586-025-02190-w 40745081

[B29] Tanner KD (2012) Promoting student metacognition. CBE Life Sci Educ 11:113–120. 10.1187/cbe.12-03-0033 22665584 PMC3366894

[B30] Tran M, et al. (2025) Situating governance and regulatory concerns for generative artificial intelligence and large language models in medical education. NPJ Digit Med 8:315. 10.1038/s41746-025-01721-z 40425695 PMC12116760

[B31] Verderame MF, Freedman VH, Kozlowski LM, McCormack WT (2018) Competency-based assessment for the training of PhD students and early-career scientists. Elife 7:e34801. 10.7554/eLife.34801 29848440 PMC6002247

[B32] Wang H, et al. (2023) Scientific discovery in the age of artificial intelligence. Nature 620:47–60. 10.1038/s41586-023-06221-2 37532811

[B33] Wei Z, Lin B-J, Chen T-W, Daie K, Svoboda K, Druckmann S (2020) A comparison of neuronal population dynamics measured with calcium imaging and electrophysiology. PLoS Comput Biol 16:e1008198. 10.1371/journal.pcbi.1008198 32931495 PMC7518847

[B34] Weidinger L, et al. (2021) Ethical and social risks of harm from language models.

[B35] Williams CG, Lee HJ, Asatsuma T, Vento-Tormo R, Haque A (2022) An introduction to spatial transcriptomics for biomedical research. Genome Med 14:68. 10.1186/s13073-022-01075-1 35761361 PMC9238181

[B36] Williams M (2018) Reagent validation to facilitate experimental reproducibility. Curr Protoc Pharmacol 81:e40. 10.1002/cpph.40 29927084

[B37] Zhang X, Van Den Pol AN (2016) Hypothalamic arcuate nucleus tyrosine hydroxylase neurons play orexigenic role in energy homeostasis. Nat Neurosci 19:1341–1347. 10.1038/nn.4372 27548245 PMC6402046

[B38] Zohar A, Barzilai S (2013) A review of research on metacognition in science education: current and future directions. Stud Sci Educ 49:121–169. 10.1080/03057267.2013.847261

