# Supplementary material for: Reflection and Experimental Rigor Are Our AiMS: A New Metacognitive Framework for Experimental Design
Source: eNeuro. 2025 Oct 24;12(10):ENEURO.0333-25.2025. doi: 10.1523/ENEURO.0333-25.2025 (PMC12571498; doi:10.1523/ENEURO.0333-25.2025)
Supplement: Figure 1-1 — Template AiMS Worksheets. Trainees and educators can download editable worksheets that guide users through the reflective prompts of the AiMS framework. Download Figure 1-1, DOCX file. [file eneuro-12-ENEURO.0333-25.2025-s002.docx]

**AiMS Framework Worksheet (Full Version)**

**(Step 0): Identify your research question**

As you prepare to articulate your experimental design, take a moment to identify the specific research question you want to solve.

- Research question:

**Step 1: Identity Components of Your Experimental System**

To address your research question, what Models, Methods, and Measurements do you plan to, or could you, use?

- Models:
- Methods:
- Measurements:

**Step 2: Examine Features of Your Experimental System**

Select one Model, Method, and Measurement that you articulated in Step 1. For each component, describe experimental parameters that map onto Specificity, Sensitivity, and Stability.

|  | **Specificity** | **Sensitivity** | **Stability** |
| --- | --- | --- | --- |
| **Model:** |  |  |  |
| **Method**: |  |  |  |
| **Measurement** |  |  |  |

**Step 3: Consider Multiple Possible Experimental Outcomes**

For at least one of your Measurements, describe multiple possible experimental outcomes you might observe. You do not yet need to describe how you might interpret each outcome.

- Measurement:
  - Possible observation #1:
  - Possible observation #2:
  - Possible observation #3:

**Step 4: Distinguish Biological Interpretation vs Technical Artifact**

For each of the possible observed outcomes that you identified, describe at least one biological interpretation of the observation and at least one technical artifact interpretation.

| **Possible Observation** | **Biological Interpretation** | **Technical Artifact Interpretation** (which needs to be ruled out through experimental design) |
| --- | --- | --- |
|  |  |  |
|  |  |  |

**(continued next page)**

**Step 5: Identify Potential Experimental Failure Points**

Identify ways in which the Model, Method, and Measurement components of your experimental system could potentially fail along the dimensions of Specificity, Sensitivity, or Stability.

|  | **Specificity - potential ways it could break/fail** | **Sensitivity - potential ways it could break/fail** | **Stability - potential ways it could break/fail** |
| --- | --- | --- | --- |
| **Model:** |  |  |  |
| **Method:** |  |  |  |
| **Measurement:** |  |  |  |

**Step 6: Plan to Monitor Performance of Your Experimental System**

Select a subset of potential technical failure modes you identified during Step 5. Brainstorm ways you could “collect data” on whether the technical failure might have occurred when you run your experiment.

| **Potential technical failure** | **Way(s) to “collect data” as to whether the experiment actually failed in this way** |
| --- | --- |
|  |  |
|  |  |

**Step 7: Identify Potential Experimental Pitfalls and Alternative Experimental Approaches**

Which of the technical limitations that you identified for your experimental system are most likely to pose technical pitfalls that are not easy to overcome through troubleshooting? Also consider other aspects of your experimental system not captured in the previous reflection exercises that may also present potential experimental pitfalls (e.g., inherently challenging experiments to execute, untested approaches). What alternative approaches could enable you to overcome the potential experimental pitfalls that you identified? Try to identify at least one alternative model, method, and measurement.

|  | **Potential Experimental Pitfall (can’t overcome)** | **Alternative Experimental Approach** |
| --- | --- | --- |
| **Model**: |  |  |
| **Method**: |  |  |
| **Measurement**: |  |  |

**Step 8: Apply a Critical Lens to Your Initial and Alternative Experimental Approaches**

- What were the primary factors driving your selection of your initial experimental Models, Methods, and Measurements?
- What factors led you to not pursue your proposed “alternative” approaches in your initial experimental design?
- Are there any advantages to trying your “alternatives” from the outset, or in parallel to your initial experimental design as a complementary approach?

**AiMS Framework Worksheet (Condensed Version)**

| **Step 1** | Model | Method | Measurement |
| --- | --- | --- | --- |
| Your research question: |  |  |  |

| **Step 2** | Specificity | Sensitivity | Stability |
| --- | --- | --- | --- |
| Model: |  |  |  |
| Method: |  |  |  |
| Measurement: |  |  |  |

| **Step 3** | Possible Observation #1 | Possible Observation #2 | Possible Observation #3 |
| --- | --- | --- | --- |
| Measurement: |  |  |  |

**Step 4**

| Possible Observation | Biological Interpretation | Technical Artifact Interpretation (which needs to be ruled out through your experimental design) |
| --- | --- | --- |
|  |  |  |
|  |  |  |

| **Step 5** | Specificity - potential ways it could break/fail | Sensitivity - potential ways it could break/fail | Stability - potential ways it could break/fail |
| --- | --- | --- | --- |
| Model: |  |  |  |
| Method: |  |  |  |
| Measurement: |  |  |  |

**Step 6**

| Potential technical failure | 3S categorization | Way(s) to “collect data” as to whether the experiment actually failed in this way |
| --- | --- | --- |
|  |  |  |
|  |  |  |

| **Step 7** | Potential Experimental Pitfall (can’t overcome) | Alternative Experimental Approach |
| --- | --- | --- |
| Model: |  |  |
| Method: |  |  |
| Measurement: |  |  |

**Step 8**

- What were the primary factors driving your selection of your initial experimental Models, Methods, and Measurements?
- What factors led you to not pursue your proposed “alternative” approaches in your initial experimental design?
- Are there any advantages to trying your “alternatives” from the outset, or in parallel to your initial experimental design as a complementary approach?
