# Supplementary material for: Reflection and Experimental Rigor Are Our AiMS: A New Metacognitive Framework for Experimental Design
Source: eNeuro. 2025 Oct 24;12(10):ENEURO.0333-25.2025. doi: 10.1523/ENEURO.0333-25.2025 (PMC12571498; doi:10.1523/ENEURO.0333-25.2025)
Supplement: Figure 1-2 — Completed AiMS Worksheet for Judy’s Neuroanatomy Case Study. This completed worksheet summarizes the neuroanatomy case study presented in the text as a concrete example of application of the AiMS framework to an experimental question and system. An editable version of the worksheet can be downloaded and modified to provide partially worked examples in an instructional context. Download Figure 1-2, DOCX file. [file eneuro-12-ENEURO.0333-25.2025-s003.docx]

**AiMS Framework Worksheet - Completed Neuroanatomy Example (Judy’s Experiment)**

| **Step 1** | *Model* | *Method* | *Measurement* |
| --- | --- | --- | --- |
| *Your research question:* To which regions of the brain do ARC-TH neurons project? | TH-Cre mouse (transgenic, not targeted knock-in) | Cre-dependent AAV-GFP viral injection into the ARC of TH-Cre mice | Detection of GFP+ fibers in brain sections |

| **Step 2** | *Specificity* | *Sensitivity* | *Stability* |
| --- | --- | --- | --- |
| Model: transgenic TH-Cre mouse | - What is the expression pattern of the Cre transgene? | - What is the expression level of the Cre transgene? | - What is the stability of the transgene over generations? - What is the consistency of transgene expression across cohorts, ages, and infected cells? |
| Method: Cre-dependent AAV-GFP viral injection into the ARC of TH-Cre mice | - What is the spread of the viral injection? - Which cell types are infected by the specific viral serotype? | - What is the titer of the virus and the effective viral load delivered to the tissue? - What is the extent of the viral infection? | - What is the viability of the virus at the time of injection? - Are virus titers consistent across batches? - Is viral gene expression consistent across animals? |
| Measurement: Detection of GFP+ fibers in brain sections | - Which microscope filters and imaging settings do I need to detect my specific fluorescent signal? - Does my imaging modality allow me to distinguish axon terminals from fibers of passage? | - What is the detection limit for fluorescently labeled axons? - What is the minimum fiber density needed to confidently identify a projection? | - What is the integrity of the tissue and fluorescent signal at the time of imaging? - To what extent are imaging and image processing settings standardized and consistent? |

| **Step 3** | *Possible Observation #1* | *Possible Observation #2* | *Possible Observation #3* | *Possible observation #4* |
| --- | --- | --- | --- | --- |
| Measurement: Detection of GFP+ fibers in brain sections to assess projection targets | Presence of GFP fibers in only previously identified target (ME), no new regions | Presence of GFP fibers in previously identified target (ME) and in PVH | Presence of GFP fibers in the ME and PVH, as well as additional brain regions | Presence of GFP fibers in the ME and additional brain regions, but not the PVH |

**Step 4**

| *Possible Observation* | *Biological Interpretation* | *Technical Artifact Interpretation (which needs to be ruled out through your experimental design)* |
| --- | --- | --- |
| Presence of GFP+ fibers in the ME but nowhere else | ARC-TH neurons project just to the known target (ME), nowhere else. This would be consistent with prior research but inconsistent with Judy’s working hypothesis. | Lack of fibers in other regions (including where the projection may only be minor) could be observed if there was poor infection of ARC-TH neurons, or if infected neurons only weakly expressed GFP. |
| GFP+ fibers observed in ME as well as in PVH | ARC-TH neurons project both to the known target (ME) and to a novel target, PVH. This would be consistent with Judy’s hypothesis. | Presence of fibers in the PVH—without ARC-TH neurons actually projecting there—could also be observed if the virus infected non-TH+ cells (*e.g.*, due to leaky Cre) or if the viral infection extended beyond the ARC to infect other cells. |
| Presence of GFP+ fibers in the ME and PVH, as well as additional brain regions | ARC-TH neurons project both to the known target (ME) and to a set of novel targets, including the PVH and other regions. This would be consistent with Judy’s hypothesis, but also gives an unexpected result. | [Same possible artifacts described in the rows above that could yield either false positive or false negative anatomical results.] |
| Presence of GFP+ fibers in the ME and additional brain regions, but not the PVH | ARC-TH neurons project both to the known target (ME) and to a set of novel targets that do not include the PVH. This would be inconsistent with Judy’s hypothesis. | [Same possible artifacts described in the rows above that could yield either false positive or false negative anatomical results.] |

| **Step 5**  Components of Experimental System | *Specificity - potential ways it could break/fail* | *Sensitivity - potential ways it could break/fail* | *Stability - potential ways it could break/fail* |
| --- | --- | --- | --- |
| Model(s): TH-Cre mouse (transgenic, not targeted knock-in) | - Transgenic Cre expression may not faithfully reflect endogenous TH expression | - Cre expression and/or activity may be insufficient to induce GFP expression in target cells | - Cre expression may change over time due to genetic drift in mouse lines (or mis-genotyping) - Cre expression may be variable across mice and litters - Cre expression may vary with age/developmental stage of mice and/or may be heterogeneous among infected cells |
| Methods: Cre-dependent AAV-GFP viral injection into the ARC of TH-Cre mice | - AAV injection might not be limited to the target brain region (ARC) - Viral serotype may preferentially infect non-neuronal cells - Since the GFP fills the entire cell, we may not be able to easily distinguish fibers of passage from axon terminals | - Virus volume, titer, or promoter activity may be insufficient for GFP expression upon Cre recombination within cells - Virus may only infect a small proportion of ARC-TH+ cells | - Virus viability may have been lost due to poor handling (e.g., kept at room temp) - Viral titers and expression levels may vary by batch and across animals - Injection volumes and/or coordinates may drift over time due to equipment issues |
| Measurement: Detection of GFP+ fibers in brain sections to assess projection targets | - May be difficult to determine sites of axon termination in brain sections - May be imaging artifacts (e.g., autofluorescence, bleedthrough) due to imaging and/or filter settings | - Fluorescent signal may be too low for microscope to detect (*i.e.,* below limit of detection) - Fiber density may be too sparse to confidently identify a projection | - Tissue integrity and fluorescent stability (*e.g.,* photo-bleaching) may degrade - Imaging and image processing settings may not be standardized or consistent across samples |

**Step 6**

| *Potential technical failure* | *Way(s) to “collect data” as to whether the experiment actually failed in this way* |
| --- | --- |
| Cre expression may not faithfully reflect TH expression | Perform double IHC for Cre and endogenous TH and evaluate overlap |
| May be insufficient virus injected for GFP expression upon Cre recombination within cells (or insufficient activity of promoter) | Analyze sections for presence of GFP in cell bodies; Repeat using viral titrations of different volumes, dilutions and/or different time courses for GFP expression and trafficking before sectioning the brain |
| Tissue integrity and/or degraded fluorescent signal may impair imaging quality | Perform additional histological analysis of tissue to assess integrity; Adjust imaging settings between different brain sections from the same animal to compare imaging quality (in case photobleaching was an issue); calibrate imaging conditions according to a standard (*e.g.*, prepared slide of fluorescent pollen) |

| **Step 7**  Components of Experimental System | *Potential Experimental Pitfall (can’t overcome)* | *Alternative Experimental Approach* |
| --- | --- | --- |
| Model: Transgenic TH-Cre mouse | If the Cre expression is not entirely restricted to TH+ neurons, this will make interpretation of the results challenging or impossible, since non-TH+ cells in the ARC have been shown to project to the PVH. | Explore whether there is a targeted knock-in mouse available, or opportunities for an intersectional approach (Cre/FLP). |
| Method: Cre-dependent AAV-GFP viral injection into the ARC of TH-Cre mice | It may not be possible to infect sufficient numbers of ARC-TH neurons while confining viral injection within the ARC, yielding incomplete/weak projection patterns. | Could complement this approach with retro AAV (rAAV) tracing, which may enable larger injection volume in different brain regions in a candidate-based approach because it doesn’t require selective labeling of projections from ARC, and then assess co-localization of rAAV-fluorophore and TH in the ARC cells. Could also use bulk injection of retrogradely transported dyes as a first-pass. |
| Measurement: Detection of GFP+ fibers in brain sections | It may be challenging to differentiate fibers of passage from synaptic terminals by imaging brain sections. | Could attempt volumetric imaging using a tissue-clearing approach or use imaging software that can reconstruct volumes from tissue sections. Alternatively, could co-label neurons with a synaptic marker like synaptophysin (*e.g.*, virally co-delivered with the fluorescent tracer in a Cre-dependent manner). |

**Step 8**

- She deemed the specific viral tracing strategy using a Cre-dependent virus injected into the ARC of TH-Cre mice to be the best approach to selectively express the GFP tracer in the dopaminergic cells of the ARC to characterize their axonal projections in a specific, yet unbiased, fashion. If she had crossed the TH-Cre mouse to a Cre-dependent GFP reporter line, she would have lost the spatial specificity and would have indiscriminately labeled all TH+ neurons, including those in other parts of the hypothalamus. She selected the specific TH-Cre transgenic mouse line based on its availability in a commercial mouse repository and because it had been previously characterized in published research. She initially chose to measure the presence of GFP+ fibers in brain sections because it seemed sufficient to answer her research question; a large body of prior neuroanatomical studies had characterized axonal projection patterns via fiber detection in brain slices.
- Judy did not initially pursue whole-mount brain imaging to characterize the pattern of ARC-TH axonal projections not only because of the precedent that imaging of brain sections would be sufficient, but also because it would require the development of novel brain clearing and imaging protocols in her lab and access to a light sheet microscope, which was not readily available in her department.
- Judy described using a retroAAV viral anatomical tracing approach—in which she would inject a Cre-dependent retroAAV at the predicted sites of ARC-TH axonal terminals to retrogradely label their cell bodies—as a potential alternative approach to her anterograde tracing strategy of injecting ARC-TH neuron cell bodies to label their axons. As a primary strategy, this approach has the limitation of being candidate-based, as opposed to unbiased, only returning information about whether ARC-TH neurons project to the specific regions where the virus was introduced. However, this would be a useful approach to validate potential target regions identified from the anterograde tracing experiments or could even be used in parallel with the anterograde tracing strategy to test Judy’s specific hypothesis that ARC-TH neurons project to the PVH.
